# A Molecular Perspective on Colistin and *Klebsiella pneumoniae*: Mode of Action, Resistance Genetics, and Phenotypic Susceptibility

**DOI:** 10.3390/diagnostics11071165

**Published:** 2021-06-25

**Authors:** Rita Elias, Aida Duarte, João Perdigão

**Affiliations:** 1 Research Institute for Medicines (iMed.ULisboa), Faculdade de Farmácia, Universidade de Lisboa, 1649-003 Lisboa, Portugal; rita.elias@tecnico.ulisboa.pt; 2 Faculdade de Farmácia, Universidade de Lisboa, 1649-003 Lisboa, Portugal; 3 Centro de Investigação Interdisciplinar Egas Moniz, Instituto Universitário Egas Moniz, Monte da Caparica, 2829-511 Almada, Portugal

**Keywords:** *Klebsiella pneumoniae*, colistin, lipid A, resistance mechanisms, susceptibility testing

## Abstract

*Klebsiella pneumoniae* is a rod-shaped, encapsulated, Gram-negative bacteria associated with multiple nosocomial infections. Multidrug-resistant (MDR) *K. pneumoniae* strains have been increasing and the therapeutic options are increasingly limited. Colistin is a long-used, polycationic, heptapeptide that has regained attention due to its activity against Gram-negative bacteria, including the MDR *K. pneumoniae* strains. However, this antibiotic has a complex mode of action that is still under research along with numerous side-effects. The acquisition of colistin resistance is mainly associated with alteration of lipid A net charge through the addition of cationic groups synthesized by the gene products of a multi-genic regulatory network. Besides mutations in these chromosomal genes, colistin resistance can also be achieved through the acquisition of plasmid-encoded genes. Nevertheless, the diversity of molecular markers for colistin resistance along with some adverse colistin properties compromises the reliability of colistin-resistance monitorization methods. The present review is focused on the colistin action and molecular resistance mechanisms, along with specific limitations on drug susceptibility testing for *K. pneumoniae*.

## 1. Introduction

Under antibiotic pressure, bacteria can rapidly evolve and develop resistance, mainly through genetic alterations in the antibiotic target genes that decrease or inhibit antimicrobial activity [1]. However, bacteria can also acquire genes encoded in genetic elements that can be mobilized via horizontal gene transfer (HGT), which eases the spread of resistance genes between different strains or species and drives the emergence of multidrug-resistant (MDR) bacteria [2,3]. MDR is defined as non-susceptibility to at least one agent in three or more antimicrobial classes [3]. Infections with MDR-bacteria are increasingly frequent and a major healthcare concern due to the limited therapeutic options that remain available. In Europe, it is estimated that more than 670,000 infections are caused by resistant bacteria from which approximately 33,000 people die per year [4].

The most frequent agents associated with life-threatening nosocomial infections and high levels of resistance reported are known as the “ESKAPE” pathogens, where ESKAPE is an acronym for *Enterococcus faecium*, *Staphylococcus aureus*, *Klebsiella pneumoniae*, *Acinetobacter baumannii*, *Pseudomonas aeruginosa*, and *Enterobacter species* [5]. Among these, *K. pneumoniae* is a rod-shaped, nonmotile Gram-negative bacteria, with a polysaccharide capsule that can be found in human skin, gastrointestinal and respiratory tracts [6,7]. According to the 2014 World Health Organization (WHO) Global Report on Surveillance of Antimicrobial Resistance, *K. pneumoniae* was considered one of the top three species causing human infections, including urinary tract infections, pneumonia, meningitis, surgical wound infections, and sepsis. In hospital-acquired pneumonia caused by *K. pneumoniae*, the mortality rate can exceed 50% in vulnerable patients, even when treated with adequate antimicrobials [7,8]. In Europe, the antimicrobial resistance surveillance for fluoroquinolone, aminoglycoside, third-generation cephalosporins and carbapenem resistance revealed that more than a third of the *K. pneumoniae* isolates (37.2%) were resistant to at least one of the previous antimicrobial classes tested, with resistant percentages ranging from 31.7% for third generation cephalosporins to 7.5% for carbapenems [7].

Considering the β-lactams, one of the antimicrobial classes most used in medicine, *K. pneumoniae* has intrinsic resistance to aminopenicillins due to a chromosomally encoded class A β-lactamase and have been acquiring other β-lactamases by HGT [9]. The two particularly worrisome ones are the extended-spectrum β-lactamases (ESBLs) and carbapenemases. ESBLs comprise bacterial enzymes that inactivate almost all β-lactams, except cephamycins, and carbapenems. Thus, bacteria with these enzymes are resistant to a wide number of antibiotics from the β-lactams class [10]. Carbapenems, on the other hand, are active and stable in the presence of ESBLs but its overuse, especially against Enterobacteriaceae infections, has been gradually acting as a selective pressure driving the emergence and increased prevalence of different enzymes with carbapenemase activity that can also be laterally mobilized by distinct mobile genetic elements [10,11]. In effect, all WHO regions have reported emerging resistance to third-generation cephalosporins and carbapenems for *K. pneumoniae* [4,8].

To cope with the increasing prevalence of MDR carbapenemase-producing Enterobacteriaceae infections, last-resort therapeutic options usually fall on drugs that are more toxic and not widely available such as colistin (polymyxin E), polymyxin B, fosfomycin, tigecycline, and aminoglycosides (e.g., gentamycin) [12,13]. Among these, colistin has been increasingly used as a last line drug to treat complicated infections by MDR carbapenemase-producing Enterobacteriaceae [14]. Also, the international consensus guidelines for the optimal use of the polymyxins clarifies that colistin is the preferred polymyxin for the treatment of lower urinary tract infections [15]. Moreover, colistin has been the subject of a renewed interest due to the emergence of molecular determinants of drug resistance that can be laterally transferred across different bacterial strains and/or species [14]. The present review is focused on colistin action and molecular resistance mechanisms, along with specific limitations on drug susceptibility testing for *K. pneumoniae*.

## 2. Colistin’s Structure and Mechanism of Action

**Figure 1:**
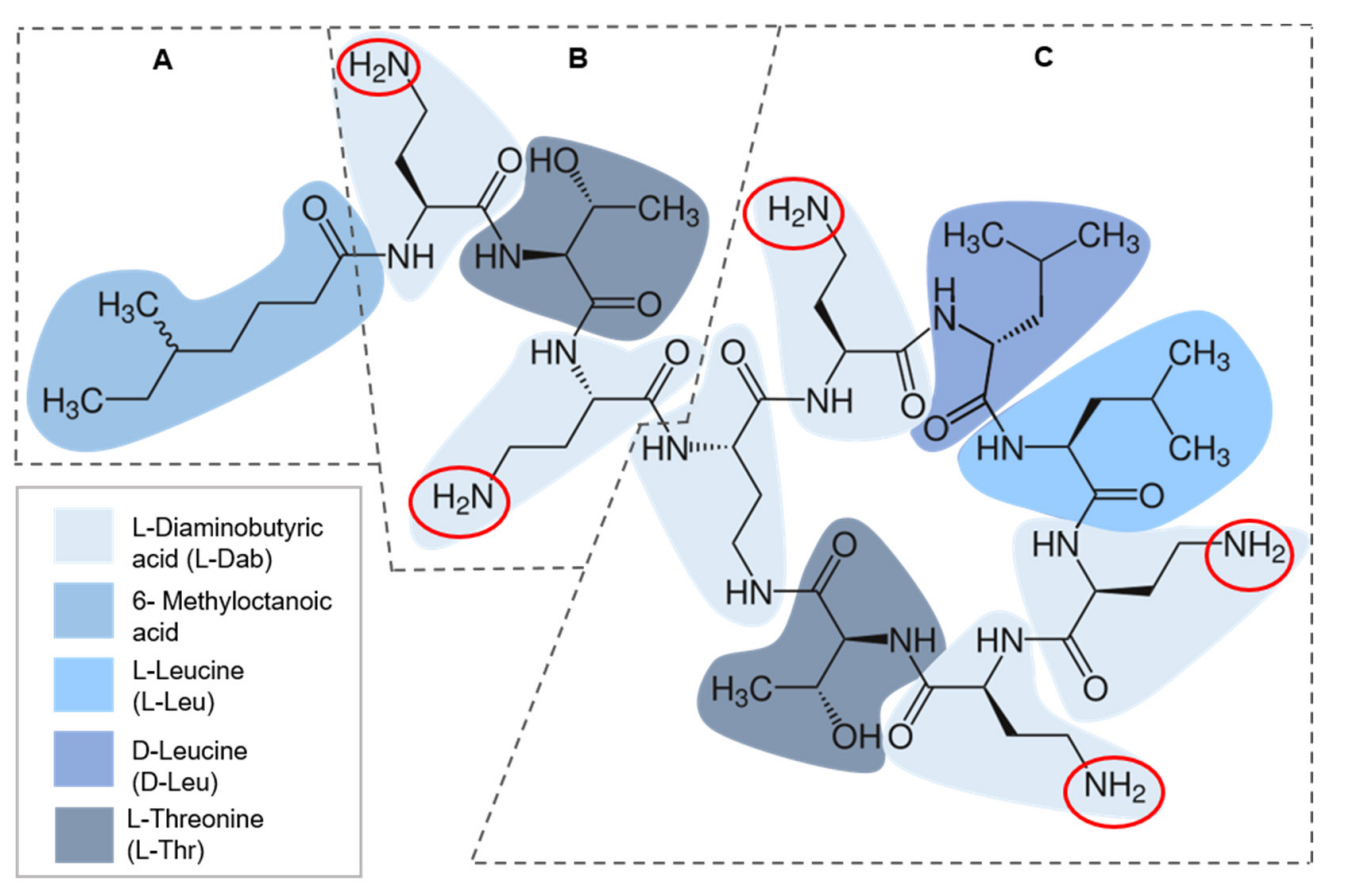
Colistin chemical structure divided in three portions: hydrophobic acyl tail segment of 6-methyloctanoic acid (**A**); linear tripeptide tail segment of diaminobutyric acid and one threonine residues (**B**); hydrophobic heptapeptide ring of diaminobutyric acid, leucine and threonine residues (**C**). Positively charged amine groups are highlighted in red [13,17,18].

Isolated from *Paenibacillus polymyxa* subsp. *colistinus*, a soil bacterium, in 1947, colistin is a cyclic polycationic heptapeptide antimicrobial belonging to the polymyxins drug class [16]. Structurally, colistin has a hydrophobic heptapeptide ring composed of four diaminobutyric acids (l-Dab), two leucine (l-Leu), and one threonine (l-Thr) residues, with three positively charged amino groups and a tail with two distinct regions (Figure 1). The first tail segment is a linear tripeptide formed by two l-Dab and one l-Thr residues, with two positively charged amino groups and the second portion is a hydrophobic acyl chain of 6-methyloctanoic acid (Figure 1). The folding of all these components originates a peculiar three-dimensional configuration that underlies its complex mechanisms of action [13,17,18].

As a bactericidal antibiotic, colistin is known to act as a detergent by attaching to the lipopolysaccharides (LPS) present in the outer membrane (OM) of Gram-negative bacteria. This attachment is determined by electrostatic interactions between colistin’s positively charged residues and the negatively charged phosphate groups of the lipid A portion of the LPS, which has an essential role in bacterial permeability control [13,17,18,19].

Under normal circumstances, lipid A negatively charged residues interact with divalent cations (calcium and magnesium) bridging adjacent LPSs to strengthen the OM and decrease its fluidity [13,17,18,19]. This interaction is also important to stabilize the LPS itself by inhibiting the electrostatic repulsion between negatively charged neighbouring LPSs. Colistin must therefore compete with these divalent cations for the binding to lipid A and, since the affinity between colistin and lipid A is at least three times higher than divalent cations and lipid A, the presence of colistin will promote the displacement of divalent cations from lipid A, which subsequently enables the interaction and binding of colistin. The latter mechanisms compromise both the LPS and OM structures and lead to the development of destabilized areas in the OM with a consequent increase in permeability. The latter also grants colistin access to the periplasmic space in what is denominated as “self-promoted uptake mechanism” [13,17,18,19]. In addition to the electrostatic interaction, colistin amphipathic nature seems to play an important role in the destabilization of the OM. It is thought that colistin can insert its hydrophobic acyl tail and the hydrophobic d-Leu^6^-l-Leu^7^ ring segment in the OM, creating holes that promote even further OM permeability [20,21,22]. Clausell et al. (2007) showed the importance of the acyl tail and the hydrophobic segment of colistin’s ring through polymyxin B analogues (polymyxin B and colistin only differ in the sixth residue of the hydrophobic ring, d-Phe^6^ and d-Leu^6^, respectively): a polymyxin B analogue deprived of acyl tail showed no antibiotic activity despite being able to bind to the LPS. On the other hand, another polymyxin B analogue with (d-Phe^6^-l-Dab^7^) instead of (d-Phe^6^-l-Leu^7^) was found to be associated with the abrogation of LPS binding due to the break of the hydrophobic domain with l-Dab substitution [22]. These important findings show that the hydrophobic domains of colistin, the acyl tail, and the d-Leu^6^-l-Leu^7^ segment, are essential to disrupt the OM, although further studies are warranted to effectively understand the specific interactions associated with colistin as well as its module-specific functions and contribution to its mode of action.

A distinct consequence associated with colistin pertains to the disruption of the inner membrane (IM). Deris et al. (2014) demonstrated that colistin can penetrate the IM and reach the cytoplasm [23], but the detailed mechanisms of this disruption that can also lead to cell death are not completely elucidated.

**Figure 2:**
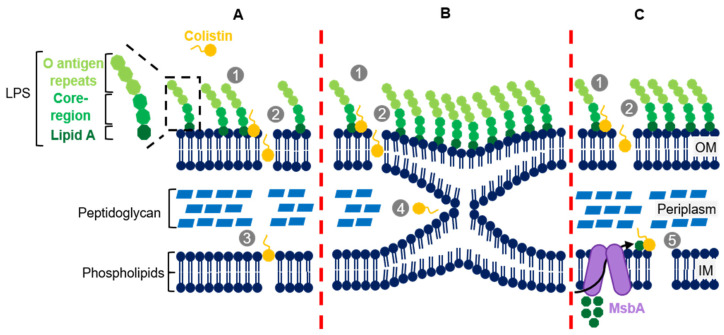
Colistin mechanisms of action: (**A**) lysis mechanism; (**B**) vesicle-vesicle contact pathway; (**C**) Inner membrane lipid A targeting. In OM, colistin induces the displacement of calcium and magnesium cations from lipid A in order to bind it (**1**). This electrostatic interaction, with the help of the hydrophobic regions of colistin, weakens the OM structure providing colistin access to periplasm (**2**). There, the way how colistin breaks IM is explained by the three hypotheses: the lysis mechanism (**A**), where colistin straddles the phospholipid bilayer decreasing the IM thickness and leading to cell lyses (**3**) [21,25]; a vesicle-vesicle contact pathway (**B**), where the colistin acyl tail induces the exchange of phospholipids between the outer leaflet of IM and inner leaflet of OM, resulting in the structure instability of theses membrane and cell death (**4**) [18,21,24]; fand, through inner membrane lipid A targeting (**C**), where colistin targets the lipid A molecules that are transiently in IM, after being translocated by MsbA from the cytoplasm (where they are synthesized) and before being transported to OM, which will induce cytoplasmic content leakage and consequently cell death (**5**) [26,27]. LPS—lipopolysaccharide; OM—outer membrane; IM—inner membrane.

One mechanism that has been proposed to underlie these findings is the vesicle-contact pathway. This pathway hypothesises that after polymyxin crosses the OM, the hydrophobic acyl tail and the cationic amino groups interact with the periplasm-facing leaflets of both the OM and IM, promoting the contact between these. This interaction would allow the exchange of phospholipids between both membranes and lead to loss of membrane integrity, osmotic pressure compromise, and cell lysis [18,21]. Clausell et al. (2006) demonstrated that a polymyxin B analogue functionally equal to polymyxin B is able to bind irreversibly to anionic vesicles, to induce the aggregation and formation of larger clusters and, if the vesicles are of monoanionic phospholipidic nature, can even induce the molecular contact and selective lipid exchange [24]. Clausell et al. (2007) have also showed, subsequently, that the cationic residues and the hydrophobic acyl tail of polymyxin B play key roles in lipid exchange: a polymyxin B analogue with two substitutions, one in position 1 and the other in position 8, by a non-charged residue (l-Dap) was significantly less effective in promoting lipid exchange (Figure 2B). Moreover, a different polymyxin B analogue without the acyl tail segment showed no ability to induce the exchange of lipids [22].

An alternative mechanism by which colistin can interfere with the physical integrity of the IM may rely on straddling the interface of phospholipid head groups and fatty acyl chains (the region where the phospholipid headgroups bound to its fatty acyl chains) leading to a decrease in IM thickness, cytoplasmic content leakage, and subsequent cell death (Figure 2A) [21,25]. Nevertheless, to our knowledge, this hypothesis has not been formally demonstrated and does not explain colistin inactivity against Gram-positive bacteria.

A more recent hypothesis on how colistin disrupts the IM is based on the lipid A biosynthesis pathway. The synthesis of lipid A begins in the cytoplasm from a UDP-N-acetylglucosamine precursor, which is submitted to a series of sequential conversions until reaching the final lipid A conformation. Then the core of the LPS is attached and the lipid A-LPS core complex is transported to the outer leaflet of IM by the MsbA transporter [26]. In parallel, oligosaccharide O is synthesized, transported to the outer leaflet of IM and only there is bound to lipid A-LPS core. The newly synthesized LPS is transported to the outer membrane by the Lpt complex where it remains attached [26]. Sabnis et al. (2020) postulated that, despite colistin bind to lipid A in OM, it might also target lipid A at the IM before it is transported to the OM. Supporting this theory, they showed the abundant presence of lipid A in the IM and also proved that the interaction between lipid A and colistin in the IM is required for colistin antibacterial activity (Figure 2C) [27]. This latter hypothesis provides a more solid base to explain the high colistin activity against Gram-negative bacteria and the absence of activity against Gram-positive microorganisms.

Besides the main mechanism of action described above, other mechanisms were found to be important. One such mechanism ensues from a modulatory effect on the host-pathogen interaction: lipid A (also known as endotoxin) induces the release of cytokines involved in shock events leading to host-cell death; however, when colistin binds to lipid A, it triggers an anti-endotoxin effect by preventing the release of these cytokines and consequently avoiding host-cell lysis [18,28]. Another mechanism, although described as secondary, is due to colistin ability to inhibit vital respiratory enzymes such as type-II NADH-quinone oxidoreductase, positioned in the IM of bacteria, interfering with the normal function of respiratory pathways [18,21].

As mentioned above, colistin activity spectrum is restricted to Gram-negative bacteria while having a more significant effect against members of the Enterobacteriaceae family including *E. coli*, *Enterobacter* spp., *Klebsiella* spp., *Citrobacter* spp., *Salmonella* spp., and *Shigella* spp. and against some non-fermentative Gram-negative bacteria like *P. aeruginosa*, *Stenotrophomonas maltophilia* and *A. baumannii*. Also, other non-Enterobacteriaceae members, *Haemophilus influenzae*, *Aeromonas* spp. and *Bordetella pertussis* are colistin susceptible [17,19]. However, *Proteus* spp. and *Serratia marcescens*, both belonging to the Enterobacteriaceae family, are naturally resistant to colistin. On the other hand, there are other non-Enterobacteriaceae species also resistant to colistin, *Morganella morganii*, *Providencia* spp., *Pseudomonas mallei*, *Burkholderia cepacia*, *Chromobacterium* spp., *Edwardsiella* spp., *Vibrio cholerae*, and *Brucella* spp. Colistin is also non-active against species from the Gram-negative cocci genus *Neisseria* [17,19].

Although colistin has a potent antibacterial activity, it is also frequently associated with side-effects, most commonly nephrotoxicity and neurotoxicity. In the 1950s (three years after colistin discovery) and the decades after, colistin sulphate and colistimethate (two available forms of colistin) were widely used in Japan and Europe to treat Gram-negative bacterial infections in humans. However, in the 1970s, due to the toxicity levels previously mentioned, its use was replaced by less toxic antibiotics such as β-lactams, aminoglycosides, and quinolones [17].

In veterinary medicine, antibiotics have been widely used for many other purposes than therapeutic, such as metaphylaxis, prophylaxis, and even for animal growth promotion, despite the fact that this practice was outlawed by the European Union in 2006 [18,29,30]. Nevertheless, since only four substances (monensin sodium, salinomycin sodium, avilamycin, and flavophospholipol) were effectively removed from the EU permitted feed additives, other antibiotics such as colistin are in use [29,30]. Even after colistin was acknowledged by WHO as a “reserve” drug for MDR infections in humans [31], the European Medicines Agency continues to approve, even though with ponderation, colistin use in animal production [32]. Thus, the misuse of colistin in animals coupled with the overuse in humans can underlie the recent emergence of colistin-resistant strains [32].

## 3. Colistin Resistance Mechanisms in *K. pneumoniae*

The clinical impact of colistin resistance has also been shown by Rojas et al. (2017), who found that colistin resistance was associated with an increased hazard for in-hospital mortality [33]. These findings lend further support to the public health threat caused by colistin resistance and stress the importance of understanding the diversity of the mechanisms underpinning resistance to this drug. Based on colistin’s mechanism of action, the most common and effective resistance mechanism is through the alteration of the lipid A negative net charge to a neutral charge, which can be accomplished by the addition of 4-amino-4-deoxy-l-arabinose (l-Ara4N), abrogating colistin binding to lipid A. The addition of phosphoethanolamine (PEtN) to lipid A comprises an alternative resistance pathway by increasing the lipid A net charge from −1.5 to −1. Although PEtN substitution is less effective than l-Ara4N at increasing the lipid A net charge, it comprises the second most common alteration underpinning colistin resistance [34,35]. In addition, lipid A hydroxylation and palmitoylation have recently been described as alternative routes to colistin resistance [35].

**Figure 3:**
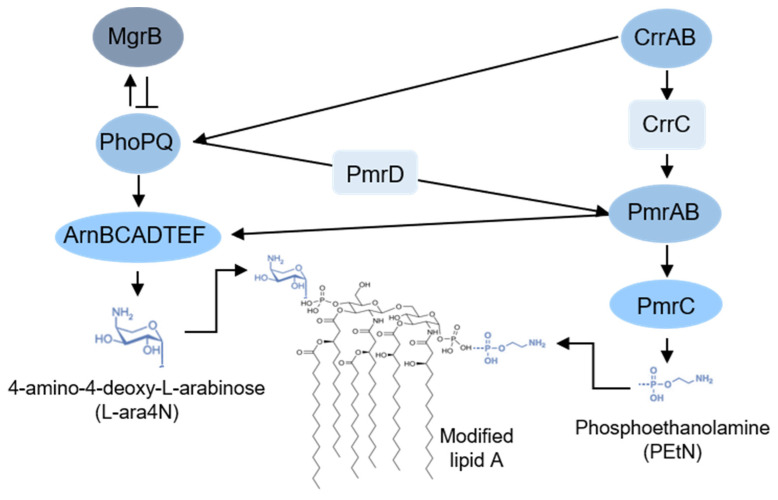
Regulatory network of Lipid A modification in *Klebsiella pneumoniae*.

The genetic basis of colistin resistance is intimately linked with complex molecular regulation mechanisms and genes involved in the LPS modification pathways. The synthesis of l-Ara4N from uridine diphosphate glucuronic acid along with l-Ara4N modification of lipid A is mediated by the gene products of the *arnBCADTEF* operon (also called *pmrHFIJKLM* operon), which is under the positive control of PhoP, the response regulator of the PhoP/PhoQ two-component system (TCS) [36,37]. The PhoP/PhoQ TCS is composed of a transmembrane sensor kinase, PhoQ, that responds to low pH, low concentration of Mg^2+^ or Ca^2+^, macrophage phagosomes or cationic antimicrobial peptides, leading to its autophosphorylation and activation of a regulator protein, PhoP, by transphosphorylation [19,34,36]. Also, this TCS is under the negative control of MgrB, a 47-amino acid transmembrane protein, which in turn is positively regulated by PhoP/PhoQ [38]. Additionally, PhoP/PhoQ can positively regulate the connector protein PmrD, which binds to PmrA inhibiting its dephosphorylation [36,39]. PmrA is the regulator protein of another important TCS, PmrA/PmrB, of which PmrB is the transmembrane sensor kinase. In the presence of high Al^3+^ or Fe^3+^, low pH or within the macrophage phagosomes, the tyrosine kinase domain of PmrB autophosphorylates and activates the regulator PmrA by transphosphorylation, which in turn can activate the transcription of the *pmrC* gene and of the *arnBCADTEF* operon. The *pmrC* gene encodes a PEtN transferase that catalyses the transfer of PEtN to lipid A [19,34,36]. In addition to this regulatory pathway, another TCS has been identified, the CrrA/CrrB. This TCS is also composed of a signal-transducing tyrosine-kinase, CrrB and a regulatory protein, CrrA. When CrrB is activated (by unknown stimuli), it will activate CrrA which acts as a positive regulator of the *crrAB*-adjacent gene *crrC.* The CrrC protein can activate the expression of both *arnBCADTEF* operon and *pmrC* gene via the PmrA/PmrB TCS (through an unknown mechanism) [40,41]. CrrB can also induce the expression of *arnBCADTEF* operon through *phoP/phoQ* but it is currently unknown if CrrC plays a role in this activation process [42] (Figure 3).

**Figure 4:**
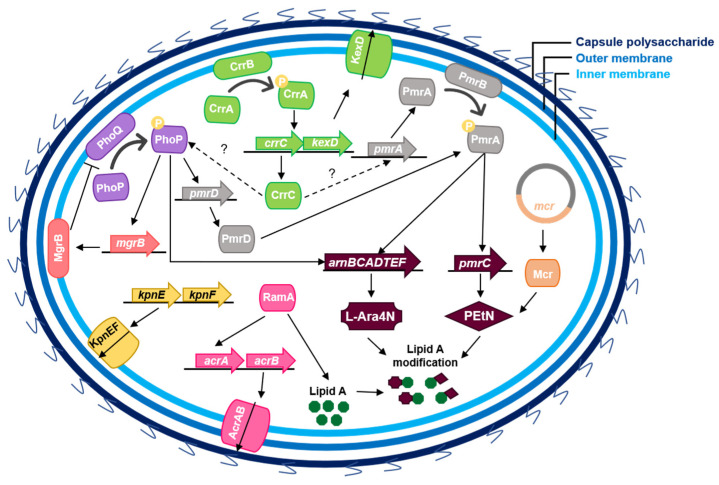
Gene regulatory and plasmid-encoding pathways of LPS modification in *Klebsiella pneumoniae*.

Therefore, the main route to achieve colistin resistance is through genetic modifications (insertions, deletions, or substitutions) in the above-described chromosomal genes. Table 1 summarizes the mutations already described in colistin resistance *K. pneumoniae* strains. However, since colistin resistance genes are only being searched in resistant isolates and the corroboration of these confirmation and/or screening of these mutations in phenotypically susceptible isolates is not usually carried out, some of these are probable phylogenetic markers that are not associated with resistance. Hence, there is a growing need regarding the knowledge on the genetic background for colistin resistance associated genes among colistin susceptible isolates. Additionally, *K. pneumoniae* can also acquire resistance through the overexpression of capsule polysaccharides, efflux pumps, or mobilization of plasmid-encoded genes (Figure 4). In fact, resistance to colistin seems to be achieved by two or more of these mechanisms [13,43]. We will next discuss these mechanisms in more detail.

### 3.1. Chromosomal Mutations Leading to LPS Modification

Since MgrB represses the PhoP/PhoQ TCS, repression/inactivation of MgrB leads to the upregulation of PhoP/PhoQ and constitutive activation of the *arnBCADTEF* operon and PmrA/PmrB via PmrD. According to diverse studies, the disruption of *mgrB* appears to be the most frequent mechanism driving colistin resistance in *K. pneumoniae* [19,34]. The disruption/inactivation of *mgrB* can be achieved by missense mutations that result in alteration on the amino acid sequence of MgrB [40,43,44,45,46,47,49,52,54], non-sense mutations that lead to premature termination of MgrB [44,47,52,55,56], insertions/deletions of nucleotide sequences in *mgrB* [43,44,46,48], and insertional inactivation by diverse families of insertion sequences either in the coding region [43,44,46,47,54,55,56,60] or in the *mgrB* promoter region [46,56,61]. Mobile genetic elements such as IS5-like, IS1 elements, and even the insertion of a carbapenem resistance element have been reported to mediate colistin resistance through insertional mutagenesis of *mgrB* [56,61]. The most frequently reported *mgrB* mutations associated with colistin resistance are the *mgrB*^Cys28Tyr^, *mgrB*^Cys28 *^, and *mgrB*^Gln30 *^ mutations (Table 1).

Mutations in PhoP/PhoQ [40,43,44,45,46,47,48,49,51,52] and PmrA/PmrB [43,44,45,46,47,48,49,50,51,52,56,57,58] TCS that lead to its constitutive activation have also been reported as associated with colistin resistance. As these TCSs control the expression of both the *arnBCADTEF* operon and *pmrC*, its constitutive activation increases the turnover of l-Ara4N and PEtN modifications to the LPS, thereby preventing the binding of colistin to its target site. The most frequent mutations reported to be associated with colistin resistance are: *phoP*^Arg114Ala^ and *phoP*^Asp191Tyr^; *phoQ*^Arg16Cys^ and *phoQ*^Asp150Gly^; *pmrA*^Gly53Cys^ and *pmrA*^Gly53Ser^*;* and *pmrB*^Thr157Pro^ and *pmrB*^Arg256Gly^ (Table 1).

Despite mutations in *mgrB*, *pmrAB*, and *phoPQ*, a total of 15 non-synonymous mutations in *crrB* gene of the CrrA/CrrB TCS were reported, with the *crrB*^Gln10Leu^, *crrB*^Asn141Tyr^, *crrB*^Pro151Leu^ and *crrB*^Gly183Val^ mutations being the most frequent (Table 1) [40,41,45,59]. In this regard, Cheng et al. (2016) demonstrated that the constitutive activation of *crrC* can be driven by *crrB* [41].

Besides the genes and TCSs described above, RamA, a global transcriptional activator from the AraC/XylS family of regulatory proteins, is involved in permeability control and multidrug resistance [17,62,63]. De Majundar et al. (2015) showed that the overexpression of *ramA* induces a decreased colistin susceptibility and that the direct binding to lipid A biosynthesis genes leads to the activation of the latter. Despite its epidemiological and clinical importance towards colistin resistance being still highly unknown, these findings suggest that RamA-dependent regulation can constitute an alternative pathway for colistin resistance [64].

Finally, mutations in the *pmrC* gene and across the *arnBCADTEF* operon have also been reported on resistant strains, but no correlation with phenotypic resistance has been established (Table 1) [43,48,49].

### 3.2. Capsule

As mentioned, *K. pneumoniae* has a lipopolysaccharide capsule surrounding its surface. It was demonstrated by Campos et al. (2004) that the upregulation of the capsular polysaccharides (CPS) synthesis has a protective role against colistin since it reduces the amount of drug that can reach the cell surface [65]. However, in the Cheng et al. (2010) study, the comparison of two strains that differ only in CPS quantity (twofold difference) showed no difference in resistance. The discrepancy between both studies was attributed to the use of *K. pneumoniae* strains with extremely low levels of CPS in the Campos study [39]. Also, it has been proposed that the release of anionic CPS by *K. pneumoniae* from its surface can trap colistin (cationic molecule). These CPSs are anchored to the LPSs which in turn are stabilized by divalent cations. The displacement of these cations as a result of colistin binding to lipid A disturb the LPS bridges promoting the release of CPS molecules, thereby inhibiting the interaction of colistin with lipid A and further reducing the amount of colistin that reaches the surface [17,66].

Capsule formation regulators also play a role in resistance namely the Rcs (regulator capsule synthesis) and the Cpx (conjugative pilus expression) [19]. LIoblet et al. (2011), showed that cross-regulatory interaction between Rcs and PhoP/PhoQ system exists but the detailed mechanism requires further studies [67]. Interestingly, Cpx appears to induce the expression of KpnEF, an efflux pump that upon deletion induces a two-fold MIC decrease [18,34,68].

### 3.3. Efflux Pumps

The expression of efflux pumps in order to pump out an antibiotic comprises a quite common resistance-mechanism for many antibiotics. In fact, efflux pumps are found not only across Gram-negative and -positive bacteria but also across eukaryotes and, consist of transporter proteins that are able to extrude a wide diversity of molecules ensuring a concentration gradient across the membrane. These transporter proteins, which bacteria have repurposed to also counteract the activity of several antimicrobial drugs, can be grouped across five superfamilies: ATP-binding cassette (ABC), major facilitator superfamily (MFS), resistance nodulation division (RND), small multidrug resistance (SMR) and, multidrug and toxic-compound extrusion (MATE) [69]. Efflux pumps across all five superfamilies have been described in *K. pneumoniae* [68,70,71,72,73] but its role in colistin resistance has been a controversial topic. Despite the mounting evidence that efflux-pump systems are involved in colistin resistance [17,34], the fact that colistin’s target is located externally suggests that, per se, the contribution of the efflux of colistin to phenotypic resistance is limited [74,75]. In fact, the efflux pump KpnEF belongs to the SMR protein family harbouring the ability to pump out some antibiotics like colistin, ceftriaxone, erythromycin, and rifampicin. Mutations in KpnEF have led to a two-fold MIC reduction for colistin and exerted a negative effect on capsule synthesis [34,68]. However, its overexpression did not lead to colistin resistance [68].

Another efflux pump described in *K. pneumoniae* is AcrAB, of the RND family, which confers multi-drug resistance to a variety of antibiotic classes. This multidrug transporter can be activated by transcriptional regulators of the AraC family, including RamA. The upregulation of *ramA* seems to induce the overexpression of AcrAB pump [62]. Naha et al. (2020) observed that the only difference between colistin susceptible and resistant *K. pneumoniae* ST147 strains was the overexpression of the AcrAB pump among one colistin resistant strain and that in the presence of the CCCP efflux pump inhibitor, a 32-fold decrease to the colistin MICs was observed for the resistant isolate, suggesting that AcrAB overexpression can be a driver of colistin resistance [76]. However, He et al. (2015) had previously described AcrAB-mediated tigecycline resistance but the same strains did not simultaneously exhibit colistin resistance [77] and, Sekyere and Aomako (2017) demonstrated that colistin MIC reduction after CCCP treatment seems to be due to the depolarization of membrane potential that when coupled with reduction of ATP levels leads to an imbalance of divalent cations favouring the binding of colistin to lipid A and, is therefore unrelated with AcrAB-mediated efflux [78].

KexD, another efflux pump from the RND-superfamily, was found to be induced in either CrrB^Asn141Ile^ or CrrB^Pro151Ser^ strains. The wild-type CrrB seems to repress *kexD*, but upon genetic alterations such as those, it is able to induce *kexD* through CrrA. However, *crrC* is simultaneously transcribed with *kexD* hampering a direct association between colistin resistance and *kexD* overexpression [42,79].

### 3.4. Plasmid-Mediated Resistance

Until 2015, colistin resistance was only described as the result of mutations in endogenous genes without the involvement of HGT. However, in November of 2015, a routine surveillance analysis of food-producing animals in China revealed strains of *E. coli* and of *K. pneumoniae* with colistin resistance phenotype not due to mutations in chromosomal genes as it was expected but due to a plasmid-encoded gene [80]. This was the first description of a plasmid-mediated gene able to confer colistin resistance and was named *mcr*-*1* [80]. This latter gene encodes the Mcr-1 protein that belongs to the PEtN transferase family and capable of mediating the transfer of PEtN to lipid A in a similar way as PmrC [19,80,81]. Since then, nine additional genes of the *mcr* family were found, *mcr-2* to *mcr-10*. Nevertheless, *mcr-1* is the most widespread mobile colistin resistance gene followed by *mcr-3*, which have, respectively, twenty-seven (*mcr-1.1* to *mcr-1.27*) and thirty (*mcr-3.1* to *mcr-3.30)* allelic variants deposited in NCBI AMR reference gene database [82,83]. Different allelic variants have also been described for the other *mcr* genes: *mcr-4* (6), *mcr-5* (4), *mcr-2* (3), *mcr-8* (3), *mcr-6* (1), *mcr-7* (1), *mcr-9* (1), and *mcr-10* (1) (Supplementary Table S1).

**Figure 5:**
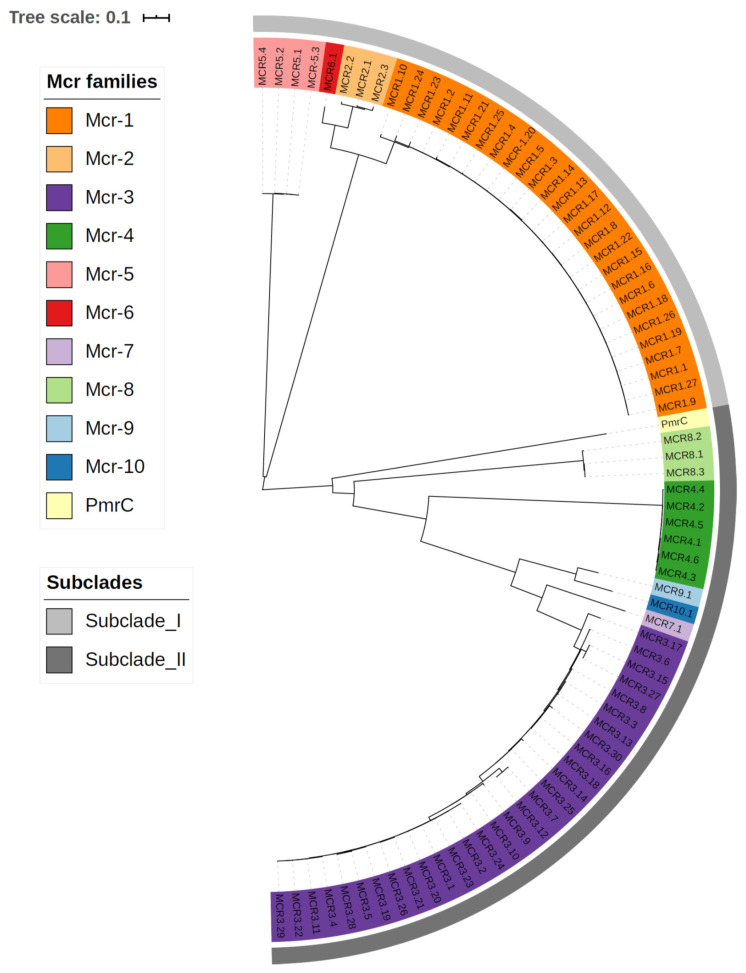
Maximum-likelihood phylogenetic tree based for the Mcr amino acid sequences (Table S1) and aligned using Seaview 4.0 [87]. The maximum-likelihood tree was obtained using PhyML as implemented in Seaview using the BLOSUM62 substitution matrix allowing for across site rate variation. The tree was annotated using the Interactive Tree of Life online tool [88].

A herein constructed phylogenetic tree based on the amino acid sequences for all Mcr alleles (Figure 5) is congruent with a topological structure across two main subclades: (i) subclade I, which includes Mcr-1, Mcr-2, Mcr-6, Mcr-5 and its allelic variants; and, (ii) subclade II that encompasses Mcr-3, Mcr-4, Mcr-7, Mcr-8, Mcr-9, Mcr-10, its variants and also PmrC. This is congruent with the findings of Xu et al. (2018) [84], Wang et al. (2020) [85], and Carrol et al. (2019) [86]. Mcr-3, Mcr-4, Mcr-7, and Mcr-9 were also found to exhibit a high degree of structural similarity that is in accordance with its placement in subclade II [86].

Presently, only a few *mcr* positive K. pneumoniae isolates were reported carrying: *mcr-1.1*, *mcr-1.2*, *mcr-1.14*, *mcr-1.15*, *mcr-2*, *mcr-3.4*, *mcr-3.20*, *mcr-3.21*, *mcr-3.22*, *mcr-3.23*, *mcr-3.26*, *mcr-3.28*, *mcr-7.1*, *mcr-8.1*, *mcr-8.2*, and *mcr-8.3* (Supplementary Table S1). Plasmids that typically harbour these genes were of the IncFII, IncI2, or IncX4 types and the *K. pneumoniae* strains reported to carry these plasmids usually belong to ST15, ST42, and ST512 [89,90,91,92,93].

## 4. Quantifying Colistin Susceptibility for *K. pneumoniae*

Despite the increasing importance of colistin as a last-resort drug along with the more recent horizontal mobilization of resistance genes, colistin susceptibility testing (CST) still raises some degree of controversy [94,95].

According to the EUCAST (European Committee on Antimicrobial Susceptibility Testing) and CLSI (Clinical and Laboratory Standard Institute), the only recommended method for colistin susceptibility testing relies upon broth microdilution (BMD) [96,97]. Dilution methodologies envisage the determination of the Minimum Inhibitory Concentration (MIC): the concentration at which no bacterial growth can be observed at the naked eye as a result of the antibiotic action [94,95]. The MIC values obtained can be then translated to resistant/susceptible phenotype according to EUCAST breakpoints: Enterobacteriaceae susceptible isolates display a MIC ≤ 2 μg/mL and resistant isolates a MIC > 2 μg/mL [98]. For this antibiotic, no Area of Technical Uncertainty (ATU) is defined [17,94,95]. Also, the CLSI-EUCAST jointly recommend the use of three quality control strains: two susceptible strains, *E. coli* ATCC 25922 (MIC = 0.25–2 μg/mL) and *Pseudomonas aeruginosa* ATCC 27853 (MIC = 0.5–4 μg/mL); and, one resistant strain, *E. coli* NCTC 13846 (MIC = 4–8 μg/mL), which harbours the *mcr-1* gene [99].

Despite BMD being the recommended method for CST, several reports have described a phenomenon designated as “skipped wells” in CST by BMD for *K. pneumoniae* isolates [33,94,95] and for other Gram-negative species, like *P. aeruginosa* [100], *A. baumannii* [101], and *Enterobacter cloacae* [102]. This phenomenon translates in an absence of growth in intermediate wells, despite the observed growth at higher concentrations [102]. CLSI has acknowledged this phenomenon and issued a recommendation for considering valid results those where one skipped well is observed in a series of colistin two-fold dilutions with the highest concentration that does not exhibit growth being the MIC value. When more than one skipped well is observed, the results should be considered as uninterpretable [103].

Although just a few reports mention the skipped wells phenomenon, it is quite frequent and lack a clear scientific basis. Turlej-Rogacka et al. (2018) observed that one out of four *K. pneumoniae* subjected to CST using the BMD method produced uninterpretable results due to skipped wells [94]. Matuschek et al. (2017) also observed this phenomenon but since they retested the strains with skipped wells until obtaining a MIC value, it is not possible to quantify the impact of this problem in this specific sample [95].

Another factor that can affect CST by BMD relies on the fact that colistin’s high affinity to plastic surfaces reduces the available colistin molecules in suspension. CLSI recommended the use of polysorbate-80 (P-80) [99,104,105,106], a surfactant, as a way to limit colistin adsorption to the plastic surface wells but later this recommendation was withdrawn due to the synergistic effect observed between both molecules and also because P-80 revealed antibacterial activity itself, especially when combined with other antimicrobials [105,106,107].

A different issue concerns the loss of colistin resistance in long-term stored isolates on glycerol-supplemented media at −70 °C [108]. Hindler and Humpries (2012) observed that among 14 isolates considered resistant in a first phase of trials, five reverted to a susceptible phenotype in the second phase of testing, after 6–8 months of storage [108]. This finding was discussed as a possible indicator of colistin heteroresistance, which has been documented in *A. baumannii* [109] and *K. pneumoniae* [108,110]. Heterorresistance is a poorly addressed topic without a uniform definition, but it is usually defined as the emergence of a resistant subpopulation within an otherwise susceptible population [53].

It is thought that the emergence of heteroresistance is related to bacterial exposure to a sub-optimal concentration of an antimicrobial drug, enabling these to explore physiological responses towards survival in the presence of that compound before a more permanent acquisition of resistance emerges and becomes fixed in the population. Nevertheless, the indiscriminate use of the “heteroresistance” term often makes the understanding of the heteroresistance clinical relevance difficult [111]. Different approaches to assess and monitor heteroresistance have been described, the most used is population analysis profiling (PAP) [109,110,112]. Other methods like disk diffusion, MIC test strip (MTS) assays, agar dilution, and flow cytometry are also used [112,113]. El-Halfawy and Valvano (2015) proposed that a first screen should be performed by disk diffusion or MTS assays and when two subpopulations can be identified, PAP should be carried out to confirm the heteroresistance phenotype [114].

The mechanisms underlying colistin heteroresistance remain poorly characterized. A 25 nt deletion in *phoP* gene has been reported to be responsible for resistance reversal in a sub-population of a resistant *K. pneumoniae* isolate due to a Asp191Tyr mutation also in *phoP* and with the heteroresistant nature of the isolate initially detected by MTS [53]. Further research to better understand its clinical relevance, definition, the best methods to monitor, and the mechanism underlying heteroresistance are highly needed.

## 5. Conclusions

Colistin is a powerful, long-used antibiotic against Gram-negative bacteria with a potent action mechanism, although quite unknown, but also with associated side-effects. The emergence of carbapenemase-producing *K. pneumoniae* strains had triggered the use of colistin to treat infections by these microorganisms. However, resistant strains have emerged too and the molecular markers underpinning drug resistance are diverse, including mutations in *mgrB*, *phoPQ*, *pmrAB*, and *crrAB* genes, the upregulation of capsule polysaccharides, the expression of efflux pumps like KpnEF, AcrAB, and KexD and the presence of plasmid-encoding *mcr* genes. These mechanisms not only warrant further investigation but also require reliable drug susceptibility testing, producing quantitative and accurate resistance levels. Additionally, innovative strategies are urgently needed to not only overcome but also prevent the emergence of resistance to this drug. The latter might include new last-resort combination therapies or novel strategies encompassing the elimination of antibiotic resistance genes or the genetic elements mediating its lateral transfer [115,116].

## Figures and Tables

**Table 1:** *Klebsiella pneumoniae* genes involved in LPS modification, gene product function, and mutations reported in the literature.

Gene	Function	Mutation ^1^	No. of Isolates Harboring the Mutation [Reference]
*phoQ*	Part of the two-component system PhoP/PhoQ that modulate the expression of *arnBCADTEF* operon, by activation of PmrA/PmrB through PmrD.	∆Lys2-Leu6	9 [43]
		Arg16Cys	1 [44]; 1 [45]
		Leu26Pro	1 [46]
		Leu96Pro	1 [47]
		Asp146Gly	5 [48]
		Asp150Gly	1 [46]; 3 [48]; 8 [49]
		Ser174Asn	1 [50]
		Val258Phe	1 [46]
		Leu348Gln	2 [47]
		Gly385Ser	1 [47]
		Ser405Arg	1 [51]
		His406Tyr	2 [51]
		Asp434Asn	1 [40]
		Val446Gly	1 [52]
*phoP*	Part of the two-component system PhoP/PhoQ that modulate the expression of *arnBCADTEF* operon, by activation of PmrA/PmrB through PmrD.	Val3Phe	1 [46]
		Leu26Gln	1 [47]
		Ser86Leu	1 [46]
		Arg114Ala	8 [49]; 7 [48]
		Arg128Ala	1 [48]
		Asp191Tyr	1 [45]; 1 [53]
*mgrB*	Encodes a small protein that negatively regulate the PhoPQ signaling system.	Val1Ala	1 [49]
		Lys2 *	1 [52]; 3 [44]
		∆Lys2-Val7	1 [43]
		Lys3 *	1 [52]
		Leu9 *	1 [47]
		∆Ala10	2 [48]
		Ile13 *	1 [47]
		Ala14Ser	2 [47]
		Trp20Arg	1 [44]
		Leu24His	1 [54]; 1 [49]
		∆Leu24Asn	1 [43]
		Val26 *	1 [47]
		Met27Lys	1 [44]
		Cys28Phe	1 [47]; 10 [43]
		Cys28Tyr	1 [40]; 1 [54]; 1 [47]; 1 [46]; 1 [52]
		Cys28Ser	37 [52]
		Cys28 *	3 [44]; 1 [47]; 1 [55]; 2 [48]
		Gln30 *	6 [44]; 1 [47]; 2 [55]; 7 [56]; 1 [52]
		Asp31Asn	1 [47]
		Phe35Ile	1 [47]
		Gly37Ser	5 [54]
		Cys39Tyr	1 [44]
		Cys39 *	2 [56]
		∆Thr40	2 [43]
		∆Ile41	2 [43]
		Asn42Tyr/Lys43lle	1 [44]; 1 [45]
		Ile45Thr	1 [44]; 1 [45]
		Pro46Ser	1 [44]
		Trp47Arg	1 [44]
		* 48Tyr	3 [46]
*pmrA*	Part of the two-component system PmrA/PmrB which modulate the expression of *arnBCADTEF* operon and *pmrC* gene which ones modifies lipid A.	Glu35Als	2 [47]
		Ser42Asn	1 [47]
		Gly53Cys	1 [47]; 1 [44]; 1 [45]
		Gly53Ser	2 [44]; 2 [45]
		Glu57Gly	1 [52]
		Ser64Thr	3 [51]
		Met66Ile	1 [51]
		Ala217Val	5 [43]; 1 [51]
*pmrC*	Catalyses the addition of a phosphoethanolamine moiety to the lipid A.	Ser25Gly	6 [43]
		Cys27Phe	3 [43]; 4 [49]; 1 [48]
		Val39Leu	7 [49]; 5 [48]
		Val42Leu	1 [49]; 3 [48]
		Leu50Val	2 [43]
		Pro135Ala	2 [43]
		Val138Ile	13 [43]
		Ala148Thr	13 [43]
		Arg152His	2 [49]; 2 [48]
		Arg155His	1 [48]
		Ser204Phe	13 [43]
		Ser257Leu	1 [49]; 2 [48]
		Ser260Leu	1 [49]; 2 [48]
		Ala279Gly	2 [49]; 5 [48]
		Gln319Arg	7 [43]; 4 [49]
		Glu354Lys	13 [43]
		Gly469Val	13 [43]
		Asp477Asn	2 [49]; 2 [48]
		Asp480Asn	1 [48]
		∆Leu521-Gly523	1 [49]
*pmrB*	Part of the two-component system PmrA/PmrB that modulate the expression of *arnBCADTEF* operon and *pmrC* gene which ones modifies lipid A.	∆Arg14	1 [50]
		Leu17Gln	1 [44]
		Leu82Arg	2 [57]
		Ser85Arg	3 [47]
		Thr140Pro	1 [47]
		Asp150His	4 [49]
		Thr157Pro	1 [43]; 2 [44]; 2 [49]; 2 [46]; 2 [50]; 1 [58]; 2 [45]; 1 [48]; 1 [52]
		Ser205Pro	2 [47]
		Ser208Asn	1 [50]
		∆Tyr209	1 [50]
		Thr246Ala	9 [43]; 4 [51]; 2 [49]; 3 [48]
		Arg256Gly	4 [43]; 4 [49]; 9 [46]; 1 [48]
		Lys280Leu	1 [46]
		Leu339Cys	1 [51]
		His340Ile	1 [51]
		Asn341Thr	1 [51]
		Arg342Asp	1 [51]
		Gln343Ser	1 [51]
		Leu344Pro	8 [49]; 7 [48]
		Pro346Gln	1 [51]
*arnA*	Bifunctional enzyme that belongs to *arnBCADTEF* operon. It promotes oxidative decarboxylation of UDP-glucuronic acid to UDP-4-keto-arabinose and the addition of a formyl group to UDP-l-Ara4N to form UDP-l-4-formamido-arabinose.	Ser18Ala	1 [48]
		Leu161Cys	1 [48]
		Thr185Ala	1 [48]
		Ile260Leu	8 [49]; 8 [48]
		Asn442Lys	8 [49]; 8 [48]
*arnB*	Belong to *arnBCADTEF* operon and catalyzes the conversion of UDP-4-keto-arabinose into UDP-4-amino-4-deoxy-l- arabinose.	Gly47Asp	2 [49]; 4 [48]
		Ala112Asp	4 [49]; 7 [48]
		Ile126Val	2 [49]; 4 [48]
		Asp285Glu	2 [49]; 3 [48]
*arnC*	Belong to *arnBCADTEF* operon and catalyses the transfer of 4-deoxy-4-formamido-l-arabinose from UDP to undecaprenyl phosphate.	Ser19Thr	2 [49]; 3 [48]
		Ser30Thr	6 [49]; 5 [48]
*arnT*	Belong to *arnBCADTEF* operon and catalyzes the transfer of the l-Ara4N moiety of the glycolipid undecaprenyl phosphate-alpha-l-Ara4N to lipid A.	Ala55Gly	8 [48]
		Ser56Leu	8 [48]
		Ala57Arg	8 [48]
		Thr58Tyr	8 [48]
		Tyr59Phe	9 [48]
		Leu114Met	1 [48]
		Ile117Val	1 [48]
		Gln156His	8 [49]
		Arg157Ser	7 [49]
		Arg158Ser	1 [49]
		Arg372Lys	6 [49]; 3 [48]
		Ile474Asn	2 [49]; 3 [48]
*arnD*	Belong to *arnBCADTEF* operon catalyses the deformylation of 4-deoxy-4-formamido-l-arabinose-phosphoundecaprenol to 4-amino-4-deoxy-l-arabinose-phosphoundecaprenol.	Trp52Leu	1 [49]
		Val53Ile	1 [49]
		Ile94Leu	4 [49]
		Ser164Pro	2 [49]
		Ile300Val	4 [49]
*crrB*	*crrB* encodes for a signal-transducing histidine kinase CrrB belonging to the CrrA/CrrB two-component system that is responsible by lipid A modification through the upregulation of PmrA/PmrB.	Gln10Leu	1 [40]; 2 [41]
		Tyr31His	1 [41]
		Ala35Val	1 [51]
		Tyr36His	1 [51]
		Phe84Ser	1 [59]
		Leu94Met	1 [40]
		Trp140Arg	1 [41]
		Asn141Ile	2 [41]
		Asn141Tyr	1 [45]; 1 [59]
		Pro151Ser	1 [41]
		Pro151Leu	1 [45]; 1 [59]
		Gly183Val	1 [45]; 1 [59]
		Ser195Asn	1 [41]
		Asn388Asp	1 [51]
		Ser379Pro	1 [51]

^1,^*—stop codon; ∆—deletion.

## Data Availability

Not applicable.

## Supplementary Materials

The following are available online at https://www.mdpi.com/article/10.3390/diagnostics11071165/s1, Table S1: List of the *mcr*-like gene families and alleles available at the NCBI AMR database and/or published, along with the GenBank accessions for the nucleotide and protein sequences found in *Klebsiella pneumoniae*.
