# Supplementary material for: A Molecular Perspective on Colistin and Klebsiella pneumoniae: Mode of Action, Resistance Genetics, and Phenotypic Susceptibility
Source: Diagnostics (Basel). 2021 Jun 25;11(7):1165. doi: 10.3390/diagnostics11071165 (PMC8305994; doi:10.3390/diagnostics11071165)
Supplement: Supplementary file 1 [file diagnostics-11-01165-s001.zip › diagnostics-1256002-supplementary.pdf]

**Supplementary Table S1** – List of the *mcr*-like gene families and alleles available at the NCBI AMR database and/or published, along with the GenBank accessions for the nucleotide and protein sequences found in *Klebsiella pneumoniae*.

| ***mcr*-gene family** | ***mcr-* allelic variants** | **Reference of the nucleotide sequence** | **Reference of the protein sequence** | **Presence in *Klebsiella pneumoniae*** |
|---|---|---|---|---|
| *mcr-1* | *mcr-1.1* | NG_050417.1 | WP_049589868.1 | Present |
| | *mcr-1.2* | NG_051170.1 | WP_065274078.1 | Present |
| | *mcr-1.3* | NG_052861.1 | WP_077064885.1 | Absent |
| | *mcr-1.4* | NG_052664.1 | WP_076611062.1 | Absent |
| | *mcr-1.5* | NG_052663.1 | WP_076611061.1 | Absent |
| | *mcr-1.6* | NG_052893.1 | WP_077248208.1 | Absent |
| | *mcr-1.7* | NG_054678.1 | WP_085562392.1 | Absent |
| | *mcr-1.8* | NG_054697.1 | WP_085562407.1 | Absent |
| | *mcr-1.9* | NG_055582.1 | WP_099982800.1 | Absent |
| | *mcr-1.10* | NG_055583.1 | WP_096807442.1 | Absent |
| | *mcr-1.11* | NG_055784.2 | WP_099982815.1 | Absent |
| | *mcr-1.12* | NG_056412.1 | WP_104009850.1 | Absent |
| | *mcr-1.13* | NG_057466.1 | WP_109545056.1 | Absent |
| | *mcr-1.14* | NG_057460.1 | WP_109545052.1 | Present |
| | *mcr-1.15* | NG_061610.1 | WP_116786830.1 | Present |
| | *mcr-1.16* | NG_064787.1 | WP_136512110.1 | Absent |
| | *mcr-1.17* | NG_064788.1 | WP_136512111.1 | Absent |
| | *mcr-1.18* | NG_064789.1 | WP_106743337.1 | Absent |
| | *mcr-1.19* | NG_065449.1 | WP_129336087.1 | Absent |
| | *mcr-1.20* | NG_065450.1 | WP_140423329.1 | Absent |
| | *mcr-1.21* | NG_065451.1 | WP_140423330.1 | Absent |
| | *mcr-1.22* | NG_065944.1 | WP_148044477.1 | Absent |
| | *mcr-1.23* | NG_067235.1 | WP_160164897.1 | Absent |
| | *mcr-1.24* | NG_067236.1 | WP_160164898.1 | Absent |
| | *mcr-1.25* | NG_067237.1 | WP_160164899.1 | Absent |
| | *mcr-1.26* | NG_068217.1 | WP_034169413.1 | Absent |
| | *mcr-1.27* | NG_068218.1 | WP_163397051.1 | Absent |
| *mcr-2* | *mcr-2.1* | NG_051171.1 | WP_065419574.1 | Present |
| | *mcr-2.2* | NG_055496.1 | WP_078254299.1 | Absent |
| | *mcr-2.3* | NG_065452.1 | WP_094323230.1 | Absent |
| *mcr-3* | *mcr-3.1* | NG_055505.1 | WP_039026394.1 | Absent |
| | *mcr-3.2* | NG_055523.1 | WP_094315354.1 | Absent |
| | *mcr-3.3* | NG_055783.1 | WP_099982814.1 | Absent |
| | *mcr-3.4* | NG_055492.1 | WP_065804663.1 | Present |
| | *mcr-3.5* | NG_055782.1 | WP_089613755.1 | Absent |
| | *mcr-3.6* | NG_055660.1 | WP_042649074.1 | Absent |
| | *mcr-3.7* | NG_055661.1 | WP_099156047.1 | Absent |
| | *mcr-3.8* | NG_055662.1 | WP_099156048.1 | Absent |
| | *mcr-3.9* | NG_055663.1 | WP_099156049.1 | Absent |
| | *mcr-3.10* | NG_055799.1 | WP_099982820.1 | Absent |
| | *mcr-3.11* | NG_056184.1 | WP_102607465.1 | Absent |
| | *mcr-3.12* | NG_057484.1 | WP_109545070.1 | Absent |
| | *mcr-3.13* | NG_060514.1 | WP_111273842.1 | Absent |

| | | | | |
|---|---|---|---|---|
| | *mcr-3.14* | NG_060515.1 | WP_111273843.1 | Absent |
| | *mcr-3.15* | NG_060516.1 | WP_111273844.1 | Absent |
| | *mcr-3.16* | NG_060517.1 | WP_111273845.1 | Absent |
| | *mcr-3.17* | NG_060518.1 | WP_111273846.1 | Absent |
| | *mcr-3.18* | NG_060519.1 | WP_111273847.1 | Absent |
| | *mcr-3.19* | NG_055497.1 | WP_087879616.1 | Absent |
| | *mcr-3.20* | NG_055493.1 | WP_065801616.1 | Present |
| | *mcr-3.21* | NG_065453.1 | WP_094312656.1 | Present |
| | *mcr-3.22* | NG_060581.2 | WP_094308975.1 | Present |
| | *mcr-3.23* | NG_060583.1 | WP_094313523.1 | Absent |
| | *mcr-3.24* | NG_060580.1 | WP_094321595.1 | Absent |
| | *mcr-3.25* | NG_060585.1 | WP_103252528.1 | Absent |
| | *mcr-3.26* | NG_065455.1 | WP_140423331.1 | Present |
| | *mcr-3.27* | NG_064790.1 | WP_017778762.1 | Absent |
| | *mcr-3.28* | NG_066546.1 | WP_150823496.1 | Present |
| | *mcr-3.29* | NG_064791.1 | WP_136512112.1 | Absent |
| | *mcr-3.30* | NG_065456.1 | WP_140423332.1 | Absent |
| *mcr-4* | *mcr-4.1* | NG_057470.1 | WP_099156046.1 | Absent |
| | *mcr-4.2* | NG_057471.1 | WP_109545058.1 | Absent |
| | *mcr-4.3* | NG_057461.1 | WP_011638903.1 | Absent |
| | *mcr-4.4* | NG_057465.1 | WP_109545055.1 | Absent |
| | *mcr-4.5* | NG_057464.1 | WP_109545054.1 | Absent |
| | *mcr-4.6* | NG_061608.1 | WP_116786828.1 | Absent |
| *mcr-5* | *mcr-5.1* | NG_055658.1 | WP_053821788.1 | Absent |
| | *mcr-5.2* | NG_057467.1 | WP_109545057.1 | Absent |
| | *mcr-5.3* | NG_061405.1 | WP_114699278.1 | Absent |
| | *mcr-5.4* | NG_065945.1 | WP_148044478.1 | Absent |
| *mcr-6* | *mcr-6.1* | NG_055781.1 | WP_099982813.1 | Absent |
| *mcr-7* | *mcr-7.1* | NG_056413.1 | WP_104009851.1 | Present |
| *mcr-8* | *mcr-8.1* | NG_061399.1 | WP_114699275.1 | Present |
| | *mcr-8.2* | NG_061627.1 | WP_072310976.1 | Present |
| | *mcr-8.3* | NG_066547.1 | WP_150823497.1 | Present |
| *mcr-9* | *mcr-9.1* | NG_064792.1 | WP_001572373.1 | Absent |
| *mcr-10* | *mcr-10.1* | NG_066767.1 | WP_023332837.1 | Absent |
